# Non-aqueous fractionation revealed changing subcellular metabolite distribution during apple fruit development

**DOI:** 10.1038/s41438-019-0178-7

**Published:** 2019-08-11

**Authors:** Wasiye F. Beshir, Takayuki Tohge, Mutsumi Watanabe, Maarten L. A. T. M. Hertog, Rainer Hoefgen, Alisdair R. Fernie, Bart M. Nicolaï

**Affiliations:** 1 Division of Mechatronics, Biostatistics and Sensors (MeBioS), Department of Biosystems (BIOSYST), KU Leuven, Leuven, Belgium; 2 Max Planck Institute of Molecular Plant Physiology (MPI-MP), Potsdam-Golm, Germany; 3 Flanders Centre of Postharvest Technology (VCBT), Leuven, Belgium

**Keywords:** Metabolomics, Plant development, Metabolism

## Abstract

In developing apple fruit, metabolic compartmentation is poorly understood due to the lack of experimental data. Distinguishing subcellular compartments in fruit using non-aqueous fractionation has been technically difficult due to the excess amount of sugars present in the different subcellular compartments limiting the resolution of the technique. The work described in this study represents the first attempt to apply non-aqueous fractionation to developing apple fruit, covering the major events occurring during fruit development (cell division, cell expansion, and maturation). Here we describe the non-aqueous fractionation method to study the subcellular compartmentation of metabolites during apple fruit development considering three main cellular compartments (cytosol, plastids, and vacuole). Evidence is presented that most of the sugars and organic acids were predominantly located in the vacuole, whereas some of the amino acids were distributed between the cytosol and the vacuole. The results showed a shift in the plastid marker from the lightest fractions in the early growth stage to the dense fractions in the later fruit growth stages. This implies that the accumulation of starch content with progressing fruit development substantially influenced the distribution of plastidial fragments within the non-aqueous density gradient applied. Results from this study provide substantial baseline information on assessing the subcellular compartmentation of metabolites in apple fruit in general and during fruit growth in particular.

## Introduction

Apple (*Malus* *×* *domestica* Borkh.) is considered an important domesticated fruit crop in temperate areas^1^, and its nutritional and economic value has been well described^2,3^. In recent years, several omics approaches have been used to study apple fruit development, including genomics, transcriptomics, proteomics, and metabolomics^4,5^. Metabolomics is increasingly used to characterize the complex physiological and biochemical changes occurring during fruit development^5–7^ and various plant stress responses^8^.

The plant primary metabolism typically takes place in different parts of the cell, with the individual metabolites showing a fast turnover and potentially being translocated between the different compartments^9,10^. One of the hurdles of metabolic studies in developing plant organs is the dilution effect by growth, at least during the early developmental stages when the volumes of the various organelles can change considerably^11^. For example, the resulting increase in vacuolar volume during cell expansion^12^ may result in underestimation of the importance of the various cytosolic or plastidic metabolites when the results are expressed on a whole-cell basis^11^. Therefore, the characterization of metabolomics in the different subcellular compartments is key to a fundamental understanding of plant functions, especially in developing apple fruit, where considerable changes in metabolite composition^5–7^ and compartmentation^12,13^ occur.

Some of the available techniques to improve the metabolite profiling at the subcellular compartment level are steady-state stable isotope labeling^14^ and non-aqueous fractionation (NAF)^15,16^. The latter method is one of the most promising approaches that allow studying a broader range of metabolite pool sizes at the subcellular level by combining GC-MS and LC-MS-based metabolomics^17–20^. NAF separates fragments of subcellular compartments and organelles in a continuous density gradient based on density differences between organelles due to differences in lipid, protein, sugars, or ionic composition^16,17^. The principle of NAF is based on the assumption that the metabolites, proteins, and other cellular components at a particular position within the cell remain stationary throughout the whole protocol^16^. Since the separation takes place using non-aqueous conditions at low temperature, the redistribution and the activities of metabolites and proteins that are present in the polar phase of the cells are prevented. Furthermore, an extensive computational analysis is required to affiliate metabolite distribution from the NAF gradient to each of the partially resolved subcellular compartments (usually plastids, cytosol, and vacuole) based on known cellular markers^21^.

The NAF method was initially applied to spinach leaves by Gerhardt and Heldt^15^ and was further developed by Stitt et al.^16^. In the past few decades, the technique has been extensively improved and applied to barley leaves^22^, maize leaves^23^, potato tubers^18^, soybean leaves^24^, and *Arabidopsis* leaves^20,25^. In addition to profiling the subcellular metabolite distributions between cytosol, plastids, and vacuole, Arrivault et al.^26^ tried to extend the method to other subcellular compartments, and successfully extended the approach to the profiling of protein subcellular distributions in *Arabidopsis* leaf material. Moreover, these authors also collected a larger number of fractions (10 to 12 fractions) from the NAF gradient than was previously done. However, these results largely confirmed earlier studies, suggesting that increasing the number of fractions does not increase compartmental resolution, as the size, the density, and the physical properties of the organelles are the limiting factor. In addition, sequential centrifugation on gradients with different density ranges for *Arabidopsis* leaf material has been reported^25^. Although the NAF technique suffers from incomplete organelle separation, it takes advantage of marker distributions to calculate subcellular distributions for the analytes of interest and with that relies on the uniqueness of the selected markers.

The work described in this study represents the first attempt to apply NAF to developing apple fruit, covering the major events occurring during fruit development (cell division, cell expansion, and maturation). The main goal of this study was to localize the metabolites in the different organelles of the cells considering the changes in metabolite distributions with progressing fruit growth. A wide range of primary and secondary metabolites was measured using GC-TOF-MS and LC-MS, respectively. The distributions of compartment-specific metabolic markers were measured and used to calculate the relevant metabolite distributions considering the three main subcellular compartments (cytosol, plastids, and vacuole).

## Results and discussion

### Flavonoid and primary metabolite changes during fruit development

**Fig. 1:**
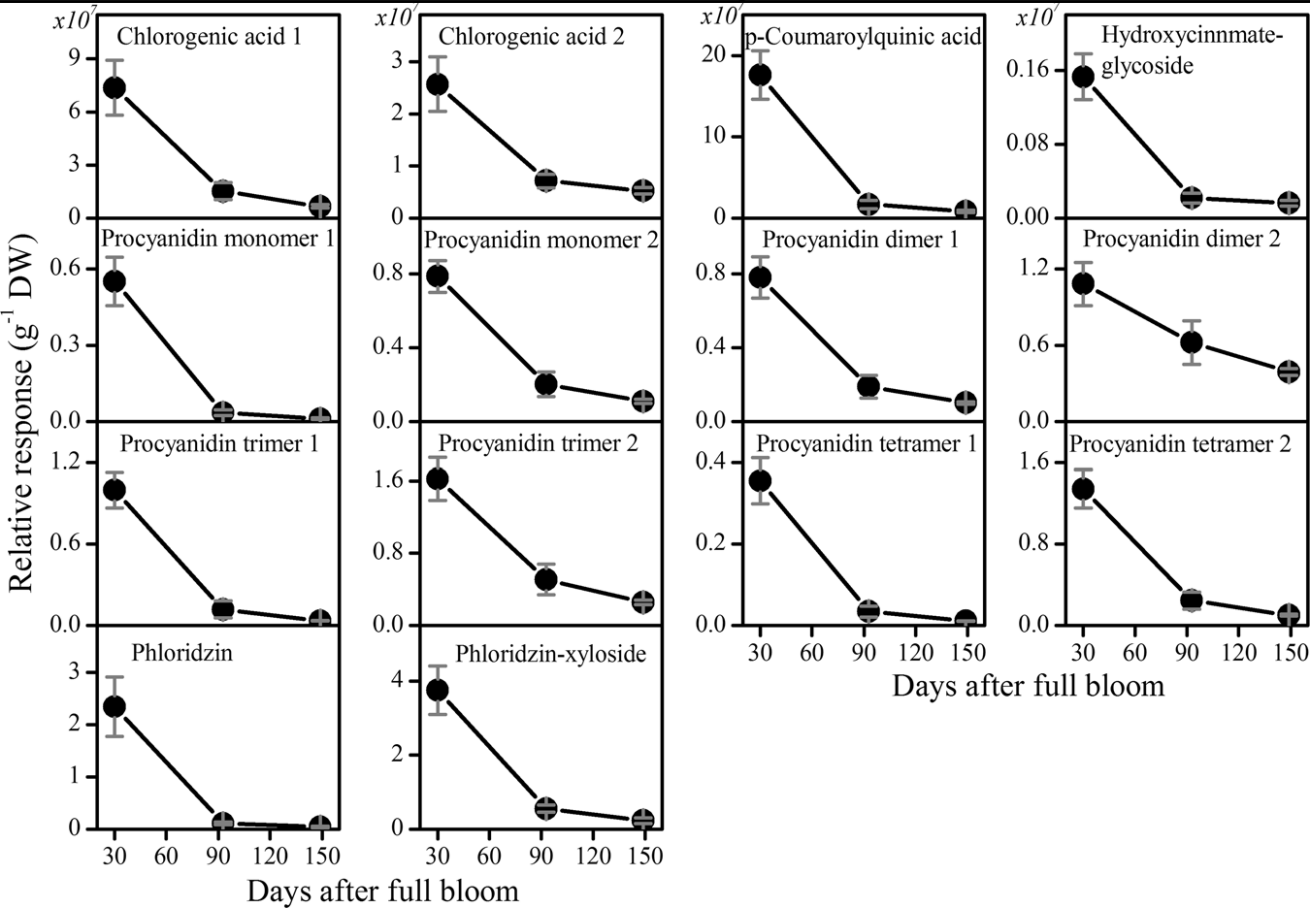
The content of flavonoids extracted from ‘Braeburn’ apple at three distinct growth stages. Metabolites were analyzed by LC-MS and expressed as relative abundance per dry weight. The fruit was harvested at three growth stages: 30, 93, and 149 days after full bloom. Values are means ± SE (*n* = 3)

**Fig. 2:**
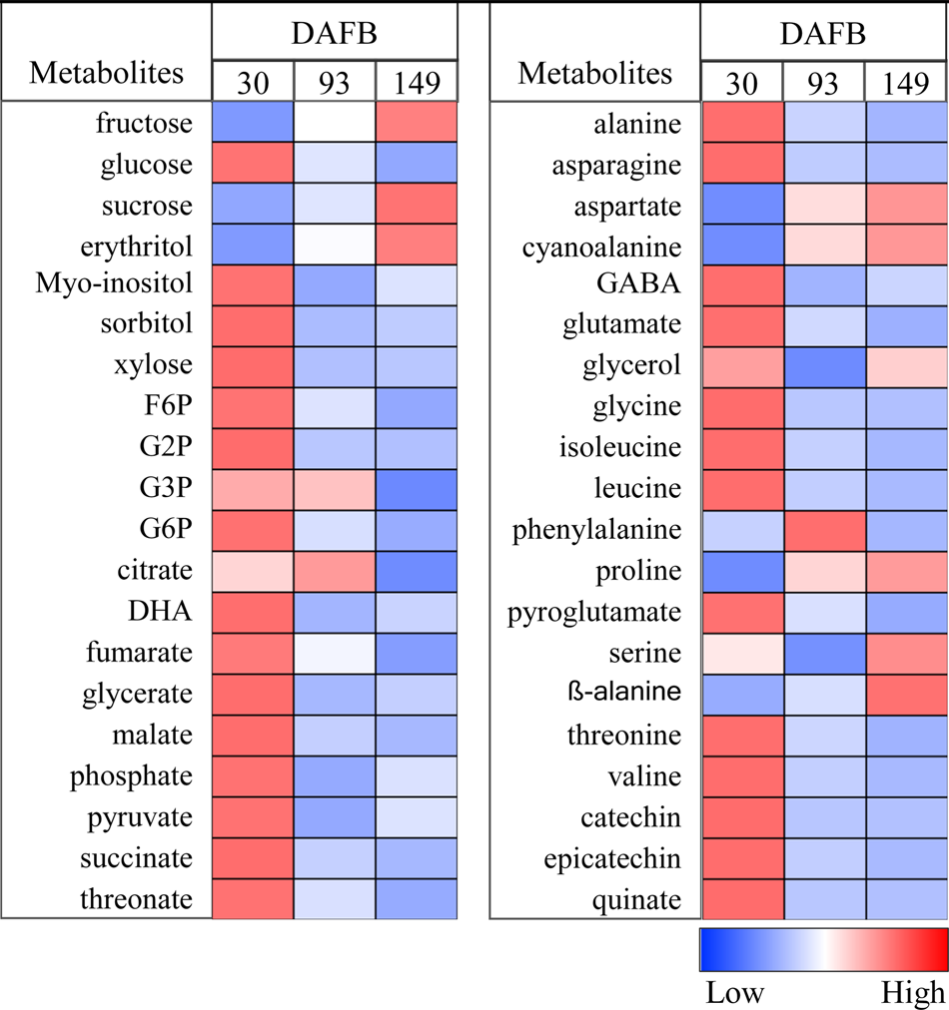
Heat maps showing the changes in primary metabolite levels during apple fruit development. The fruit was harvested at three growth stages: 30, 93, and 149 days after full bloom. The colors were generated from GC-MS metabolite profiling raw data (relative value) after mean center and scale transformation into comparable levels. The light blue color denotes a lower concentration of metabolites, whereas the deep red color denotes a higher concentration of metabolites. Values are means ± SE (*n* = 3). Heat maps were generated using the MultiExperiment Viewer software (MeV v4.9.0, http://www.tm4.org/)^32^. DHA dehydroascorbate, F6P fructose 6-phosphate, G2P glucose 2-phosphate, G3P glucose 3-phosphate, G6P glucose 6-phosphate, GABA γ-aminobutyrate

Based on the analyses of the complete tissue samples before fractionation, the general trends of the changing flavonoids and primary metabolite levels during the course of fruit growth are summarized in Figs. 1 and 2.

Flavonoids represent one of the largest classes of plant secondary metabolites, comprising different subgroups, including proanthocyanidins, anthocyanins, and flavonols. The flavonoids family analyzed by LC-MS comprised two peaks of chlorogenic acid (5-caffeoylquinic acid), *p*-coumaroylquinic acid, hydroxycinnamate-glycoside, phloridzin, phloridzin-xyloside, and six unknown procyanidins (comprising dimer I & II, trimer I & II, and tetramer I & II; Fig. 1). In addition to the flavonoids analyzed by LC-MS, quinate, epicatechin, and catechin, as well as chlorogenic acid, were analyzed by GC-MS together with the main primary metabolites. The results from the harvested apple fruit showed that the levels of these different classes of flavonoids increased during cell division and substantially declined throughout the fruit developmental continuum (Fig. 1). In addition, apple shows a higher amount of phloretin and phloridzin than any other plant species^27,28^, although the physiological relevance of phloridzin to the plant is not understood yet. The changes in flavonoid content observed during fruit development were in good agreement with the data of other apple cultivars^5,6,29^.

The relative concentrations of sucrose and fructose, the most abundant sugars present in mature apple fruit, on a dry weight basis were low in the early growth stages and substantially increased toward fruit maturation (Fig. 2). Unlike fructose and sucrose, the relative contribution on a dry weight basis of glucose, *myo*-inositol, sorbitol, and xylose, including sugar phosphates and most organic acids, were very high at 30 d and declined at 93 and 149 d (Fig. 2). In addition, most of the free amino acids present in apple fruit were high at 30 d on a dry weight basis, which coincides with a higher rate of protein synthesis during cell division^30,31^, and declined during the latter growth stages (Fig. 2). In contrast, aspartate, ß-alanine, cyanoalanine, and proline showed higher values at 149 d (Fig. 2). Moreover, we have shown earlier the changes in cellular primary metabolite levels during apple fruit development, which covered five distinct growth stages^7^. The metabolome results obtained in this earlier study confirmed these previous metabolome data in developing apple fruit^5,6^. The current study focuses instead on the subcellular metabolite distributions^32^.

### Separation of subcellular compartments of growing apple fruit

The separation of subcellular compartments using the NAF technique was done using the method described in ref. ^18^ for potato tubers. A linear NAF gradient of 1430–1620 kg m^−3^ (C_2_Cl_4_/C_7_H_16_ mixture), used for potato tubers, resulted during the initial trial runs in most of the cellular material, being focused in the top few fractions and showing limited enrichment in the lower three fractions of the gradient (Supplementary Fig. S1). Therefore, the NAF gradient and centrifugation speed and duration were altered to achieve a wider distribution of the cellular material over the whole NAF gradient. After a series of experiments, the best distribution of the cellular material was achieved using a linear gradient range of 1300–1620 kg m^−3^ centrifuged at 25,000 × *g* for 2.5 h. This gradient is comparable to the various non-aqueous density gradients reported to fractionate plant material, for example: 1280−1510 kg m^−3^ with a cushion at 1620 kg m^−3^ for spinach leaves^21^, 1320−1500 kg m^−3^ with a cushion at 1560 kg m^−3^ for barley leaves^22^, 1250−1590 kg m^−3^ for maize leaves^23^, although, as mentioned above, considerably broader than the 1430−1620 kg m^−3^, with a cushion at 1620 kg m^−3^ for potato tubers^18^ and *Arabidopsis* leaves^20,25^.

**Fig. 3:**
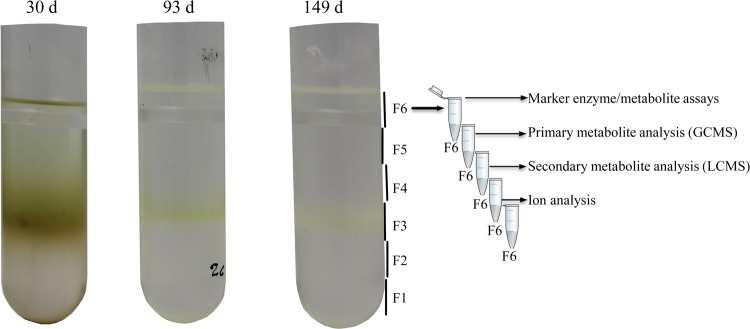
A schematic representation of the NAF gradients, sampling of the gradient, and subsequent analysis of marker enzymes and different class of metabolites. The three gradients represent the three distinct growth stages of apple fruit development at 30, 93, and 149 d after full bloom. A total of six fractions (F1 to F6) were considered in this study. Each fraction was aliquoted and used for various marker assays, primary and secondary metabolite analysis, and ion analysis

Each gradient was divided into six fractions for further analysis, as outlined in Fig. 3. A band was observed in the middle area of the NAF gradient (Fig. 3, fraction 3), which was in line with the expectations. As demonstrated for potato tuber samples, this band was likely due to the presence of a large amount of starch^17,18^. Due to this band formation, a higher amount of plant material was recovered from the middle fraction (F3) than from the other fractions, saturating the instrumental analysis with high abundant metabolites such as glucose, fructose, sucrose, sorbitol, and malate. For this reason, smaller sample volumes were analyzed for fraction F3, and the results were corrected accordingly.

### Compartment-specific markers for developing apple fruit

**Fig. 4:**
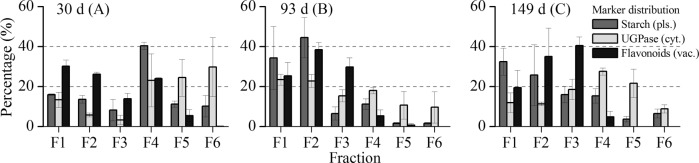
Compartment-specific marker distributions in NAF gradient from three growth stages of apple fruit (30, 93, and 149 d after full bloom). The distribution of plastidic (starch, gray), cytosolic (UGPase, white), and vacuolar (flavonoids, black) markers is shown as the average of three independent biological replicates expressed as a percent of the total (±SE). The mean distribution of flavonoids was used as a vacuolar marker

Figure 4 shows the distribution of compartment-specific markers in the NAF gradient for the three growth stages of apple fruit. Initially, AGPase was targeted as a plastidial marker. However, its activity was very low in the fractioned material and gave less reliable data. For this reason, starch, as it is synthesized and stored in semi-crystalline granules inside the plastids^18,33^, was considered as a plastidial marker. The distribution of starch in the fractioned material showed systematic changes associated with the changing tissue structure and metabolic composition of the cell throughout fruit development. In the early growth stage (30 d), starch was predominantly located in the fourth fraction, with a mean ± SE concentration of 40.4 ± 1.8%, whereas in the later growth stages it was mainly found in the dense fractions (F1 and F2), which may reflect developmental differences. It can be speculated that the accumulation of starch with progressing fruit development resulted in more densely packed starch granules—as indicated by the chloro-chromo-amyloplast functional transition of plastids^34^—increasing their density. Schaeffer et al.^34^ reported a significant increase in the size of plastids throughout the course of apple fruit development and observed a significant increase in the percentage of the plastids occupied by starch, as evident by the overall area of starch granules and accompanied by plastoglobuli and other sub-organellar structural changes.

As for the vacuolar marker, flavonoids are synthesized on the endoplasmic reticulum and exclusively accumulated in the vacuole of many plant species^3,13,35,36^. Krueger et al.^20^ showed that the subcellular distribution of flavonoids in NAF gradient for *Arabidopsis* shows a similar trend as the vacuolar marker nitrate and can, therefore, be used as a vacuolar marker. Flavonoids were found in the lower part of the gradient, showing little to zero relative enrichment in the lightest fractions (Fig. 4). Unlike most of the flavonoids, the distribution of phloridzin followed a deviant trend as it was mainly found in the lightest fractions of the gradient. The current results suggest that phloridzin was partially associated with the plastidial and cytosolic markers. This could reflect the synthesis and storage of this specific flavonoid, as also Yamaki^13^ reported some of the phenolic compounds to be additionally found in low levels in the apoplast and the cytosol of young growing apple fruit. For these reasons, phloridzin was excluded from the list of flavonoids used as a vacuolar marker. The cytosolic marker UGPase was evenly distributed throughout the fractions in agreement with previous reports^18,20,21^, irrespective of the growth stage.

In general, the marker distributions of the fractioned apple tissue found in this study are comparable to results on developing potato tubers^18^ and partially comparable to results on spinach leaves^21^ although the values revealed large standard deviations. In contrast, a clearly resolved vacuolar marker enrichment in the densest fraction, F1, reported for *Arabidopsis thaliana* leaf tissue^20,26^ was not observed in the current study. This finding might be due to a difference in subcellular composition between apple fruit cortical tissue on one hand and *Arabidopsis* leaf tissue on the other; the vacuoles of apple fruit are used to store reserve soluble carbohydrates as well as polyphenols and organic acids^13^, whereas the vacuole in *Arabidopsis* leaf material mainly accumulates mineral deposits and proteins^37^.

### Reproducibility of the non-aqueous fractionation fractions

**Fig. 5:**
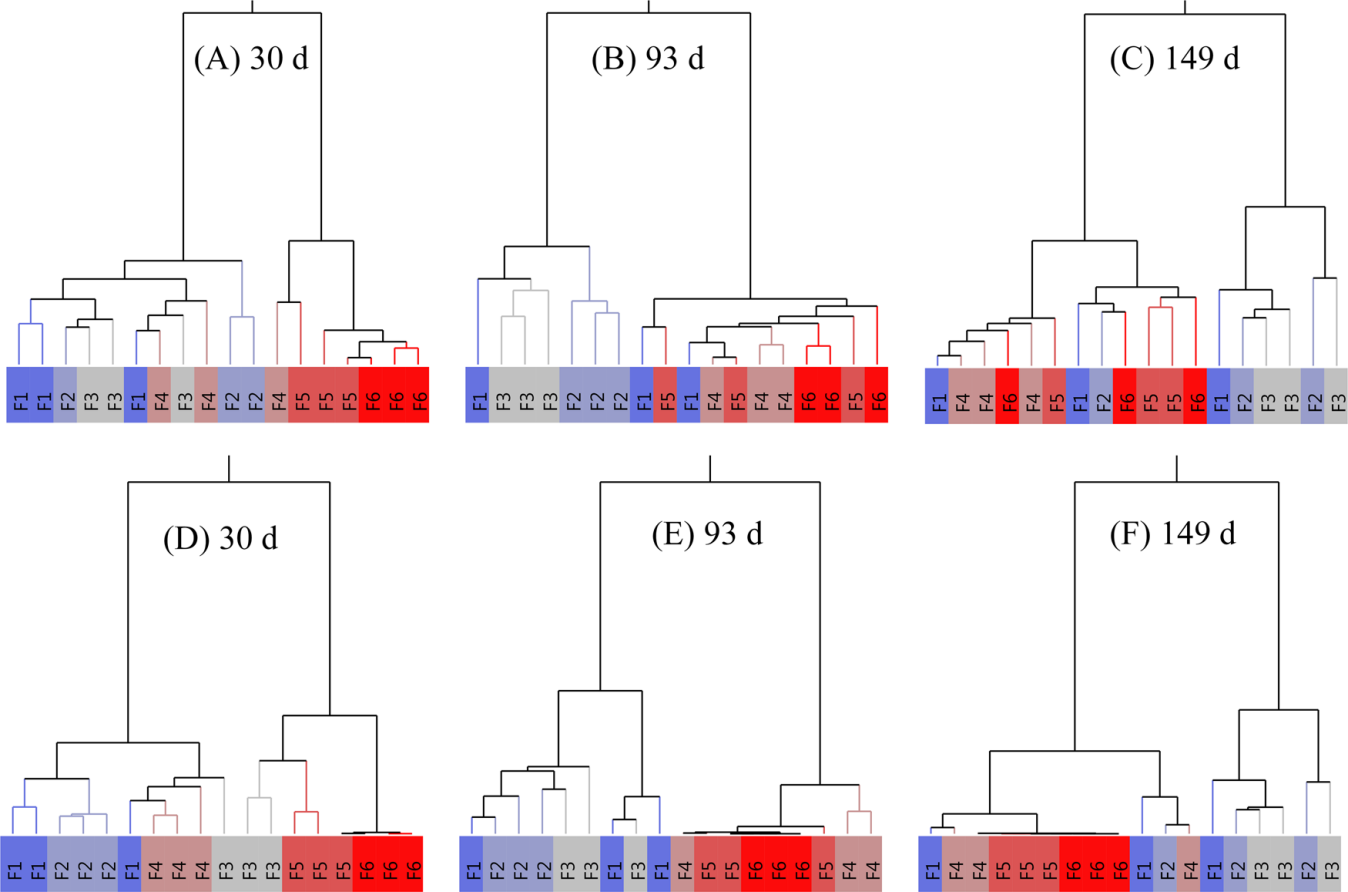
Hierarchical cluster analysis (HCA) of metabolite data along a density gradient. HCA plot was performed for the three independent replicates based on the scaled data, considering the distribution of either primary (A─C) or secondary (D─F) metabolites between the six fractions. The analysis was performed for the three developmental stages of the fruit (30, 93, and 149 d after full bloom). The deep blue (dense fraction F1) to deep red (light fraction F6) colors represent the density gradient of the replicated six fractions. In the heat map, identical gradient fractions are encoded with the same color

To further investigate the general reliability of the NAF technique, hierarchical cluster analysis (HCA) was performed for the three independent replicates based on the scaled data, considering the distribution of primary (Fig. 5a–c) and secondary (Fig. 5d–f) metabolites between the six fractions. The analysis was performed for the three developmental stages of the fruit (30, 93, and 149 d after full bloom). To evaluate the performance of the clustering the normalized mutual information (nmi) was calculated^38^; a nmi score of 1 indicated a perfect clustering of the 6 fractions into 6 clusters, while a score of 0 indicated completely incorrect clustering. Depending on the number of clusters considered, the performance of the clustering changes. As an example, in the case of the secondary metabolites at 96 d, a perfect subdivision of the six fractions over two clusters was observed (resulting in the theoretically maximum possible nmi of 0.622), but by considering 6 clusters the replicate fractions are not correctly grouped, as indicated by an nmi of 0.654, which theoretically could have been 1.

At 30 d, when cell division was mostly active^4^, the three independent replicates of the six fractions, based on both the primary and secondary metabolites, showed the best clustering considering 6 clusters (Fig. 5a, d) with the highest nmi values (respectively 0.678 and 0.7825 given a theoretical maximum value of 1). This indicated that for young fruit the different fractions were more unique in their metabolic composition, enabling a better clustering. For the next growth stage at 93 d (Fig. 5b, e) the grouping in two clusters proved to perform best, showing the highest nmi values (respectively 0.5138 and 0.622 given a theoretical maximum value of 0.622). This indicated that the vacuolar enriched fractions (F1-F3) were clearly separated from the top three fractions (F4−F6), but within these two clusters metabolic differences were limited. At the final growth stage of 149 d, the fractions were less well clustered, with lower and top fractions becoming mixed (Fig. 5c, f), resulting in relatively low nmi values considering two clusters (respectively 0.3937 and 0.3973 given a theoretical maximum value of 0.622). This is most likely because the plastidial and vacuolar compartments were interfering more at this later growth stage, as shown in Fig. 4. In addition, the principal component analysis (PCA) plot shown in Supplementary Fig. S2 supports the HCA distribution for both primary and secondary metabolite data. The decreasing ability to cluster the replicate fractions with increasing growth stage might be due to the increasing biological variation between individual fruit depending on aspects like the position of the fruit within the tree. While at a young stage (30 d) fruit might be more similar, over time spatial variation will tend to accumulate, increasing the variation between the replicate fruit and making it more difficult to obtain highly repeatable fractionations during NAF.

### Subcellular distribution of primary metabolites

**Fig. 6:**
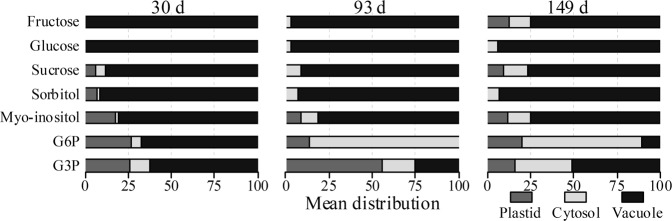
Subcellular distribution of selected sugar, sugar alcohol, and sugar-phosphate metabolites of apple fruit harvested at three distinct growth stages (30, 93, and 149 d after full bloom). Metabolite levels are expressed as percentages. The different subcellular compartments are indicated by the gray (plastid), light gray (cytosol), and black (vacuole) colored bars

The subcellular compartmentation of the major classes of metabolites including sugars, sugar alcohols and phosphates, amino and organic acids and phenolic compounds of apple fruit harvested at three distinct growth stages (30, 93, and 149 d after full bloom) is summarized in Table 1. The metabolite content in each subcellular compartment is presented as a percentage of the total amount present in the tissue. Most of the metabolites were accumulated in the vacuole to a greater extent than in the cytosol and plastid. For instance, sorbitol and sucrose were dominantly located in the vacuole (Fig. 6). In developing apple fruit sorbitol and sucrose are the two main photosynthates produced in the leaves and translocated into the sink fruit^39^. As a result, the apoplast, the sieve tube, and the cell wall space, which serve as bridges between the source leaves and sink fruit, are expected to contain a considerable portion of these metabolites. It should be noted that, therefore, the distribution of sorbitol and sucrose could not unequivocally have been marked as exclusively cytosolic, plastidial, or vacuolar. In support of this, Zhang et al.^40^ reported about 930 mM total soluble sugars in the cytosol of growing apple fruit, whereas the apoplast content was higher than 400 mM. In addition, Yamaki^13^ found about 40–50% of the sucrose and sorbitol content was located in the extracellular space, whereas more than 95% of glucose and fructose was found in the intracellular space. Similarly, a higher amount of sucrose was found in the sieve tube of spinach leaves as compared to the cytosol^21^.

**Table 1:** Subcellular distribution of selected metabolites of ‘Braeburn’ apple fruit harvested at three distinct growth stages (30, 93, and 149 d after full bloom)

Metabolites	Subcellular distribution [%]								
	30 d after full bloom			93 d after full bloom			149 d after full bloom		
	Plastid	Cytosol	Vacuole	Plastid	Cytosol	Vacuole	Plastid	Cytosol	Vacuole
*Carbohydrates*									
Sucrose	6	6	88	0	9	91	9	14	77
Sorbitol	7	1	92	0	7	93	0	7	93
Fructose	0	0	100	0	3	97	13	12	75
Glucose	0	0	100	0	3	97	0	6	94
Myo-inositol	18	2	80	8	11	81	12	13	75
G3P	26	11	63	55	19	26	16	33	51
G6P	27	5	68	13	87	0	20	70	11
*Organic acids*									
Pyruvate	24	1	75	9	15	76	28	14	58
Citrate	32	5	63	0	26	74	0	12	88
Succinate	10	0	90	11	4	85	5	4	92
Fumarate	5	3	92	20	0	80	16	0	84
Malate	0	0	100	0	4	96	0	4	96
*Amino acids*									
Alanine	10	0	90	26	12	62	0	14	86
Asparagine	14	0	85	7	3	90	23	1	77
Aspartate	1	0	99	5	6	89	7	0	93
Beta-alanine	10	16	74	7	43	51	0	26	74
GABA	5	0	95	27	1	71	16	0	84
Glutamate	25	0	75	0	33	67	0	0	100
Glycine	7	27	67	3	21	76	10	15	75
Isoleucine	9	57	34	0	100	0	6	92	2
Leucine	14	2	84	27	2	71	0	17	83
Phenylalanine	13	55	33	0	100	0	6	91	3
Proline	10	2	89	18	14	68	5	21	74
Serine	10	0	90	2	1	97	4	12	84
Valine	7	46	47	5	95	0	9	88	4

Metabolite levels are expressed as a percentage in the different subcellular compartments

After performing the NAF data analysis, about 92% of sorbitol was associated with the vacuolar marker. In the later growth stages, sorbitol followed a distribution similar to the early growth stage. In contrast, in the early two growth stages, nearly 90% of sucrose was located in the vacuole as compared to 77% at 149 d. The latter comes close to values of about 77% for sucrose and glucose being located in the vacuole of potato tubers^18^. In mature fruit, sucrose showed a shift from the vacuole into the cytosol and plastids, comprising respectively 14 and 9% of the cellular sucrose. Such a decline could be associated with sucrose being mobilized from the vacuole to the cytosol to supplement the energy used to fuel the increasing cellular respiration during the final stage of fruit growth, following the onset of the climacteric rise at 149 d^7^. In addition, the increase in the cytosolic percentage of sucrose is in line with the higher synthesis of sucrose possible through sucrose phosphate synthase activity which leads to sucrose accumulation during ripening^31,41^. Despite the lack of evidence on whether sucrose can be synthesized or imported into the plastid, there are several studies that describe its presence in plastids^18,33,42^. In a comparison between isolated protoplasts and vacuoles from growing apple fruit (harvested at 30−50 g fresh weight) sorbitol seems to be located completely in the vacuole, whereas only 10% of sucrose was located in the vacuole^13^. These results are in agreement with the current sorbitol data, but inconsistent with the high sucrose levels, we observed in the vacuole. However, Arrivault et al.^26^ have demonstrated that the extracellular compartment showed protein distributions similar to that of the vacuole whilst studies using crystalline cellulose as a marker for the apoplast came to the same conclusion^43^, indicating that these compartments are effectively not well separated. The currently observed high sucrose levels might, therefore, rather be related to the extracellular space instead of to the vacuole.

The presence of sucrose in the plastid compartment was accompanied by 13% of the cellular fructose. Additionally, glucose showed a pronounced accumulation in the vacuole, whereas going from being non-detectable at 30 d to approximately 3 and 6% cytosolic distribution at 93 and 149 d, respectively. Both fructose and glucose were predominantly localized in the vacuole of the cells showing a similar distribution in the early two growth stages. Although glucose was largely assigned to the vacuole, the actual concentrations could be higher in the cytosol or the plastids due to their smaller size^18^. For instance, Yamaki^13^ reported about 80% of the protoplast in growing apple fruit is occupied by the vacuole. Similarly, it has been estimated that the vacuole in 10 weeks old growing potato tubers occupies about 67% of the cellular volume whilst cytosol and plastids occupy 12 and 15%, respectively^18^.

For all three growth stages, most of the sugar alcohols, including ribitol and erythritol, were located in the vacuole, whilst about 20% of *myo*-inositol was distributed between cytosol and plastid (Fig. 6). Phosphate sugars were predominantly localized in the cytosol at 93 and 149 d (Fig. 6). However, it is worth noting that substantial amounts of G6P and G3P were found in the vacuole, particularly in the early stage of development (30 d). The presence of phosphorylated compounds in the vacuolar compartment is unlikely given the fact that these compounds are known to be exclusively located in the cytosol and plastids^36^. Previous NAF-studies on developing potato tubers^18,33^ found hexose phosphates and glycolytic intermediates mainly distributed between the plastid and cytosol. However, claims have been made for the presence of up to 34% of G1P correlated with the vacuolar marker^18,33^. These authors suggested that the variability in the MS measurement for metabolites that are present in very low concentration, as was the case for most sugar phosphates, might influence the calculated relative subcellular distributions of these metabolites.

It should be noted that a considerable amount of the major tricarboxylic acid (TCA) cycle intermediates such as succinate, fumarate, and malate accumulated in the vacuole. Malate is known to be a major storage compound in apple fruit^6^, with an increase in its content as the vacuoles increase in size with cell expansion^11,12^. At 30 d, malate is completely localized in the vacuole, whilst at 93 and 149 d, about 96% was located in the vacuole^13^. This vacuolar malate accumulating during fruit growth may serve as an alternative substrate to fuel cellular respiration^12^ during postharvest storage when sugars become depleted. In spinach leaves, Gerhardt and Heldt^15^ observed a similar distribution of malate with the vacuolar marker. Apparently, succinate and fumarate are also stored in the vacuole of apple cells, although 10 to 20% of the total fumarate and succinate was found in the cytosol and plastid compartment. Despite the clear separation of the TCA cycle intermediates between the three compartments (plastid, cytosol, and vacuole), several studies reported that the mitochondria cannot be unambiguously separated from the cytosol using the NAF technique and most likely are included as part of the cytosolic fraction^16,18–20,26^. The cytosol compartment was, therefore, considered to contain the mitochondria compartment as well. In line with this, pyruvate showed a higher relative abundance in the cytosol and plastid with 25, 24, and 42% at 30, 93, and 149 d, respectively. In this study, the relative distributions of most of the amino acids were similar to the organic acids distributions. However, isoleucine, phenylalanine, and valine were mainly contained in the cytosol and plastid of apple cell.

### Subcellular distribution of free ions

**Fig. 7:**
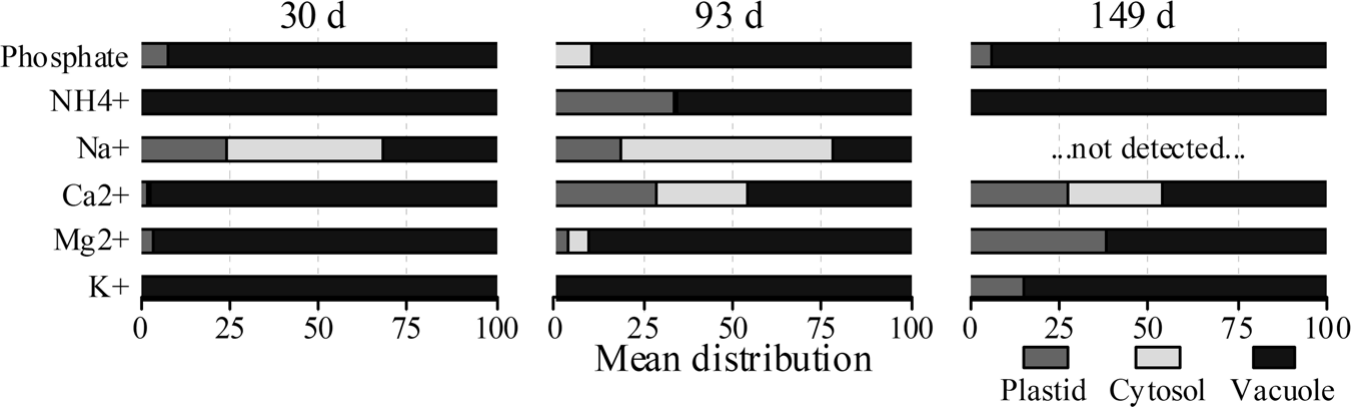
Subcellular distribution of ions of apple fruit harvested at three distinct growth stages (30, 93, and 149 d after full bloom) measured using ion chromatography. Ion levels are expressed as percentage. The different subcellular compartments are indicated by the gray (plastid), light gray (cytosol), and black (vacuole) colored bars

Non-aqueous fractionation has been used to assess the subcellular distribution of both primary and secondary metabolites^15,16,18,20^ and specific proteins^26^. In the current study, different free ions were analyzed using ion chromatography in the fractionated material; these have been reported to reside mainly in the tonoplast and vacuole of plant cells^36^. Several cations including K^+^, Mg^2+^, Ca^2+^, Na^+^, and NH_4_^+^, as well as other anion and organic acids including phosphate, citrate, and one additional peak combining malate and succinate were detected. As shown in Fig. 7, most of the cations and phosphate were predominantly located in the vacuole of apple cortical tissue. In contrast, at 30 and 93 d, sodium was mainly distributed in the cytosol, plastids, and vacuole compartments, while its value was below the detection limit in mature fruit (at 149 d). The vacuole, which is known to store phosphate^44^, indeed contained over 95% of the phosphate irrespective of the growth stage (Fig. 7). Phosphate is required for the biosynthesis of numerous P-containing compounds and can be remobilized from the vacuole^44^.

## Conclusions

In conclusion, we effectively implemented the NAF technique for apple tissue by establishing a density gradient between 1300–1620 kg m^−3^ and applying a centrifugation at 25,000 × *g* for 2.5 h. The plastidial marker showed a shift from the lightest fractions in the early growth stage to the dense fractions in the later fruit growth stages. This implies that the accumulation of starch content with progressing fruit development substantially contributed to the subcellular distribution of plastidial fragments during non-aqueous density gradient fractionation. The results showed that most of the sugars and organic acids such as malic acid were predominantly located in the vacuole, whereas some of the amino acids were distributed between the vacuole and the cytosol.

In accordance with literature results, similar results were obtained for flavonoids and sugars levels during fruit growth. For example, flavonoid accumulation occurred at the early stages of fruit development, whereas sucrose and fructose—the most abundant sugars present in mature apple fruit—were low during the early stages of fruit life and substantially increased toward fruit maturation. In contrast, the relative concentration of glucose was high at 30 d and declined during cell expansion and maturation. Moreover, understanding compartmented pools of metabolites during fruit development will help us to improve our understanding of the regulation of cellular metabolism during fruit development, which is fundamental to the quality of the harvested fruit.

## Materials and methods

### Plant materials and chemicals

Developing ‘Braeburn’ apple fruit were collected from 2-year-old ‘Braeburn’ trees grafted on M9 rootstock, growing at the KU Leuven experimental orchard in Rillaar, Belgium (50°57′48.8″N, 4°52′56.5″E). The fruit was harvested throughout the 2015−2016 growing season at three distinct growth stages; 30, 93, and 149 d after full bloom, covering cell division, cell expansion, and the onset of the climacteric rise. Tissue samples were collected from the cortical hypanthium tissue, immediately frozen in liquid nitrogen and stored at −80 °C. Samples were transported on dry ice to the Max Planck Institute of Molecular Plant Physiology (MPI-MP), Potsdam-Golm, Germany, where the analyses were carried out. In addition, all chemicals were purchased from either Merck or Sigma (Darmstadt, Germany) and stored according to the manufacturer’s instructions.

### Non-aqueous fractionation experimental procedures

The frozen hypanthium tissue samples were homogenized using a Mixer Mill (Retsch, MM 400, Haan, Germany) at a frequency of 25 Hz for 2 min. About 5 g fresh weight homogenized apple tissue was lyophilized at 3 Pa for 96 h. The freeze dryer was precooled to −80 °C. About 500 mg lyophilized material was suspended in 20 mL anhydrous C_2_Cl_4_/C_7_H_16_ mixture (66:34, v/v). To avoid contamination by condensed water during the NAF procedures, the organic solvents were dried and stored on molecule sieve beads with 0.3 nm pore size. The suspension was ultrasonicated for 2 min (with 6 × 10 cycles at 65% power). The sonicated suspension was filtered through a 40-µm nylon net to remove bigger particles. After centrifugation for 10 min at 3200 × *g* and 4 °C, the supernatant was discarded, and the pellet was suspended in 3 mL C_2_Cl_4_/C_7_H_16_ mixture (66:34, v/v). This suspension was loaded on an NAF gradient (ranging from 1300 kg m^−3^ to 1620 kg m^−3^ of 32 mL C_2_Cl_4_/C_7_H_16_ mixture) and subjected to density gradient centrifugation (25,000 × *g* for 2.5 h at 4 °C). The gradient was divided into six fractions, which were each subdivided into five equal aliquots for primary metabolite analysis, secondary metabolite analysis, marker analysis, and free ion analysis. To each aliquot 1 mL of heptane was added and centrifuged for 3 min at 4 °C. The suspension was discarded, and the pellet was dried for 6 h using a vacuum concentrator operating at room temperature (SpeedVac concentrator, Thermo, Waltham, MA). Each NAF experiment was performed three times starting from independent biological plant material. The results were used to characterize compartment-specific markers, and metabolite distribution was expressed on a dry weight basis.

### Profiling of primary metabolites

Extraction and derivatization of primary metabolites were done by a method modified from Lisec et al.^45^. As ribitol is present in apple fruit in trace amounts^46^, it was replaced as an internal standard by phenyl-ß-D-glucopyranoside. Polar metabolites were extracted by adding 700 μL of 80% methanol to the NAF fractions. A stainless-steel ball (2 mm diameter) was added to each gradient fraction to enhance homogenization of the gradient fractions suspension. The solution was incubated in a thermomixer (Eppendorf AG, Hamburg, Germany) at 70 °C for 15 min with shaking at 1400 rpm. Then, 300 μL of H_2_O was added, and the extract was centrifuged at 22,000 *g* at 4 °C for 20 min. Finally, 400 μL of the supernatant was dried by speed vacuum drying at room temperature.

Metabolites were derivatized, combining oximation and silylation. First, 60 μL of methoxyamine hydrochloride (20 g/L in anhydrous pyridine) was transferred into an Eppendorf tube containing the dried sample and incubated in a thermomixer at 37 °C for 2 h. Subsequently, 120 μL of MSTFA (*N*-Methyl-*N*-(trimethylsilyl) trifluoroacetamide) was added to the mixture and incubated at 37 °C during 30 min while shaking at 1400 rpm. Then, the samples were centrifuged (5417 R centrifuge, Eppendorf AG, Germany) at 22,000 × *g* for 3 min at 20 °C to obtain clear supernatant. Finally, 100 μL of the derivatized sample was transferred into glass vials containing deactivated glass inserts and analyzed using GC-TOF-MS.

Metabolite data acquisition was performed by using the Pegasus HT-C for GC-TOF-MS. Analysis of GC-MS was performed as described previously by Lisec et al.^45^. Metabolite annotation was done using TagFinder^47^ and Xcalibur 2.1 software by matching the acquired spectra against the Golm metabolome database (GMD) and the NIST library (National Institute of Standards and Technology, Gaithersburg, USA).

### Secondary metabolic analysis

Secondary metabolites were extracted using 200 μL of 80% methanol, by shaking on a Mixer Mill at a frequency of 25 Hz for 2 min. Glass beads were added into the vials to homogenize the lyophilized plant materials. The suspension was centrifuged for 10 min at 22,000 × *g* at 4 °C. Then, the supernatant was transferred to a new 1.5-mL Eppendorf tube and centrifuged for 5 min at 22,000 × *g* at 4 °C. Finally, 70 μL of the supernatant was transferred into glass vials with deactivated inserts.

Secondary metabolites were measured using a surveyor high-performance liquid chromatography (HPLC) system coupled with Finnigan LTQ-XP system (both Thermo Finnigan, USA). Analysis of LC-MS was performed as described previously by Tohge and Fernie^48^. All data were processed using Xcalibur 2.1 software (Thermo Fisher Scientific, Waltham, USA). Metabolite identification and annotation were performed using standard compounds and metabolomics database^49^.

### Extraction and analysis of compartment-specific markers

Extracts for assay of enzymes were obtained as described by Geigenberger and Stitt^50^. Analysis of ADP glucose pyrophosphorylase (AGPase, E.C. 2.2.7.27) and UDP glucose pyrophosphorylase (UGPase; E.C. 2.7.7.9) was performed as described by Gibon et al.^51^ and Zrenner et al.^52^, respectively.

Starch was assayed as previously described by Hendriks et al.^53^. Soluble sugars of the pellet obtained after the extraction of polar metabolites were further extracted with 80% ethanol at 70 °C for 15 min, followed by washing the pellets with 1 mL of water. The pellets were dried and homogenized in 0.2 mM KOH and incubated for 1 h by heating them at 95 °C. After acidification to pH 4.9 with 1 M acetic acid/sodium-acetate buffer, the suspension was digested overnight with a mixture of amyloglucosidase and α-amylase. The starch content of the sample was calculated from the glucose content of the supernatant. The glucose content was determined from the NADPH formed during the enzymatic reactions, spectrophotometrically by measuring its absorbance at 340 nm.

### Ion chromatography

Plant extracts (50 μL) with 80% methanol for LC-MS analysis were vacuum-dried and then dissolved in 550 μL of LC-MS grade water. Ions were quantified by ion chromatography (Dionex ICS‐3000; Dionex, Idstein, Germany) with a KOH gradient for anions and with a methanesulfonic acid gradient for cations following the manufacturer’s instructions. Data were processed using CHROMELEON v.6.8 software (Dionex). A standard curve for each ion was used to determine the corresponding ion concentration in the samples.

### Data analysis

The evaluation of the percentage abundance of cellular constitutes in each of the resolved compartments was done by the *BestFit* command line tool, version 1.2 (http://csbdb.de/csbdb/bestfit/bestfit.html)^19,20^. The evaluation was done by testing all possible subcellular distributions for a given metabolite applying 1% intervals, thus generating 5151 possible distribution given the three compartments^21^. HCA and PCA were done using the JMP software, version 13.0 (SAS Institute Inc., Cary, NC, USA). To evaluate the performance of the clustering, the normalized mutual information was calculated in Matlab (The MathWorks, Inc., Natick, MA, USA) using the NMI function^38^.

## Supplementary information

supplementary materials
