# Supplementary material for: Non-aqueous fractionation revealed changing subcellular metabolite distribution during apple fruit development
Source: Hortic Res. 2019 Aug 11;6:98. doi: 10.1038/s41438-019-0178-7 (PMC6804870; doi:10.1038/s41438-019-0178-7)
Supplement: Supplementary file 1 — supplementary materials [file 41438_2019_178_MOESM1_ESM.docx]

Non-aqueous fractionation revealed changing subcellular metabolite distributions during apple fruit development

**Wasiye F. Beshir^1^, Takayuki Tohge^2^, Mutsumi Watanabe^2^, Maarten L.A.T.M. Hertog^1^, Rainer Hoefgen^2^, Alisdair R. Fernie^2^, Bart M. Nicolaï^1,3*^**

^1^Division of Mechatronics, Biostatistics and Sensors (MeBioS), department of Biosystems (BIOSYST), KU Leuven, Leuven, Belgium.

^2^Max Planck Institute of Molecular Plant Physiology (MPI-MP), Potsdam-Golm, Germany,

^3^Flanders Centre of Postharvest Technology (VCBT), Leuven, Belgium.

*Correspondence: Prof. Bart Nicolai ([bart.nicolai@kuleuven.be](mailto:bart.nicolai@kuleuven.be))

# Supplementary material


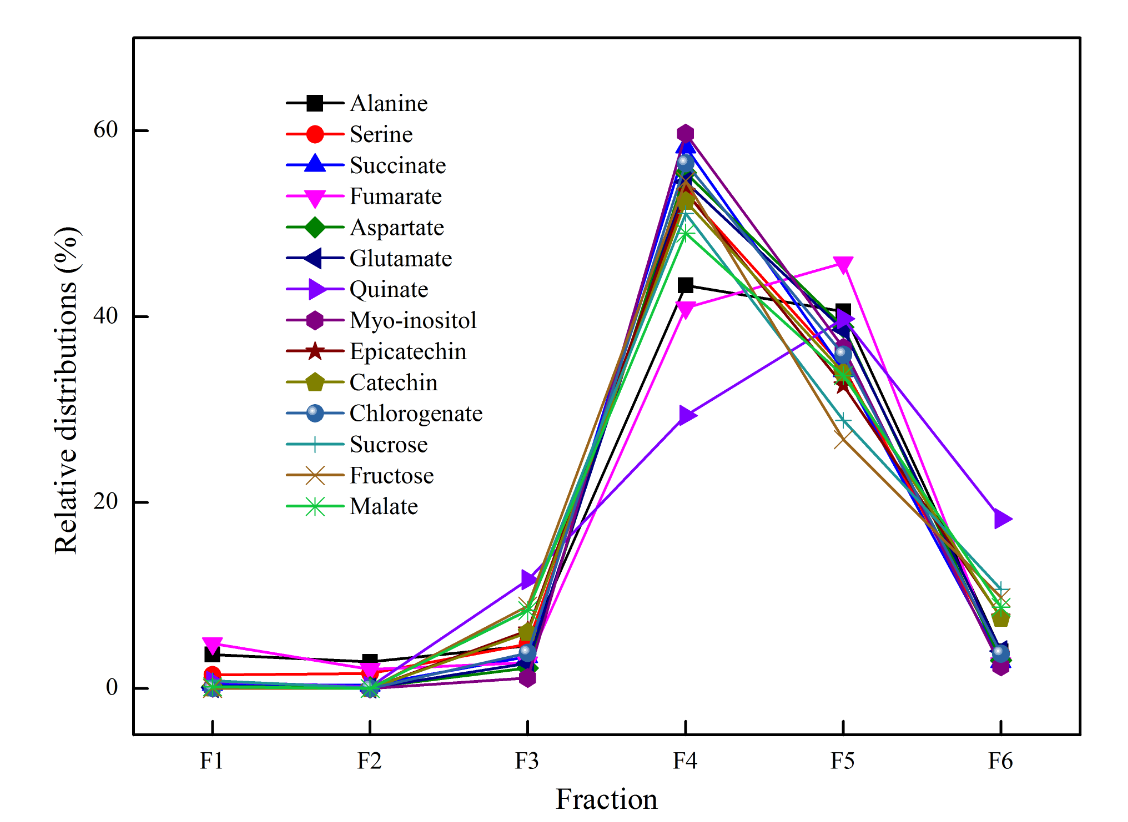
Fig. S1: Distribution of selected metabolites of apple fruit harvested at 30 d after full bloom across the NAF-gradient. A linear NAF-gradient of 1.43−1.62 kg m^-3^ (C_2_Cl_4_/C_7_H_16_ mixture) used previously for *Arabidopsis thaliana* leaf tissue, resulted in of the cellular material focused in the top few fractions (fractions F4−F6).


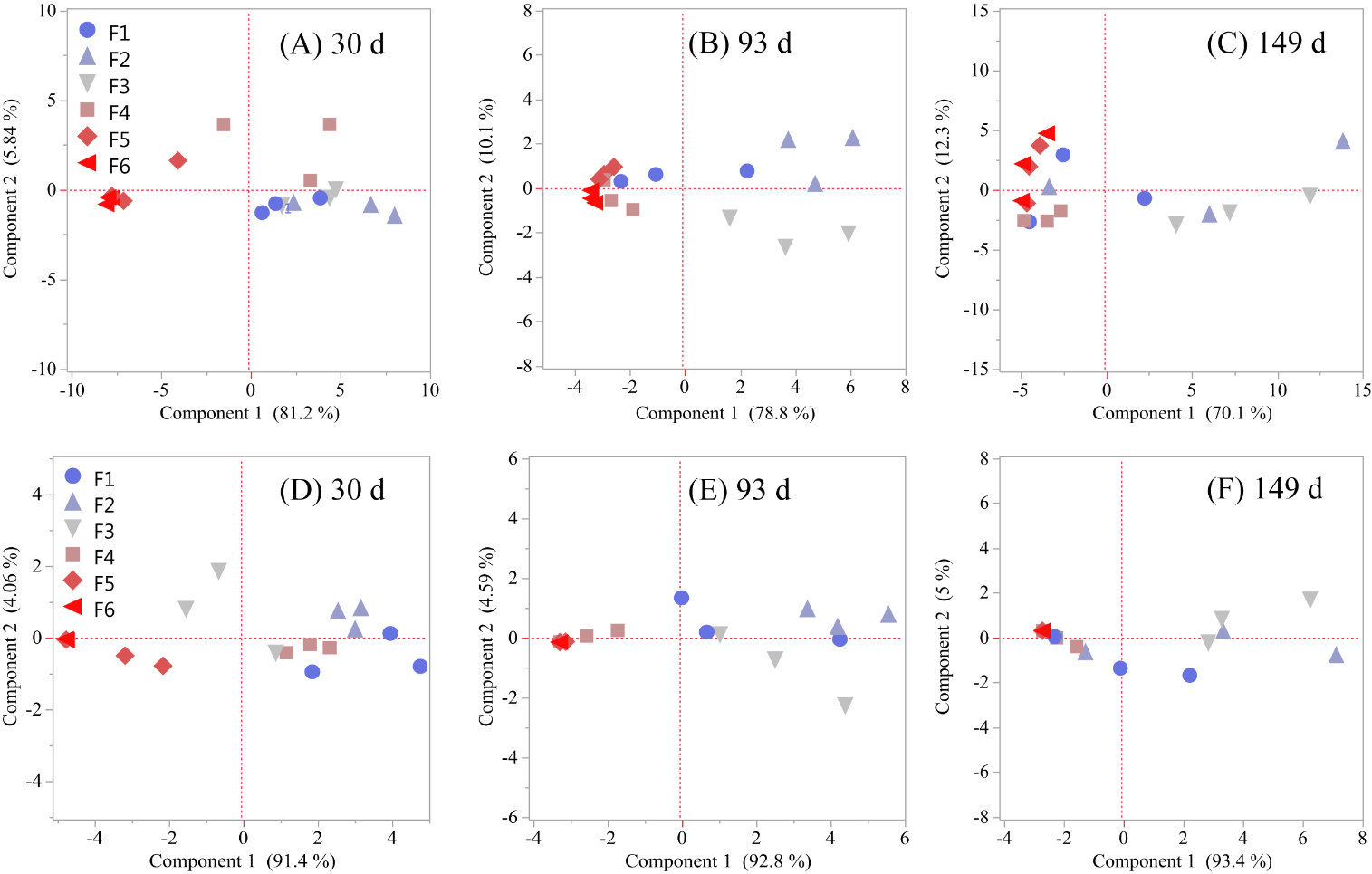
Fig. S2: Principal component analysis (PCA) of primary (A─C) and secondary (D─F) metabolite data along a density gradient. PCA plot was performed for the three independent replicates based on the scaled data, considering the distribution of individual metabolites between the six fractions. The analysis was performed for the three developmental stages of the fruit (30 d, 93 d, and 149 d after full bloom). The deep blue (dense fraction F1) to the deep red (light fraction F6) represents the six fractions with respect to a density gradient.
